# Case Report: Unusual Aggregation of Different Glomerulopathies in a Family Resolved by Genetic Testing and Reverse Phenotyping

**DOI:** 10.3389/fped.2022.826330

**Published:** 2022-02-28

**Authors:** Reeti Kumar, Vahakn Keskinyan, Megan Chryst Stangl, Brandon M. Lane, Anne F. Buckley, Laura Barisoni, David N. Howell, Rasheed A. Gbadegesin

**Affiliations:** ^1^Division of Nephrology, Department of Pediatrics, Duke University Medical Center, Durham, NC, United States; ^2^Division of Renal Pathology, Department of Pathology, Duke University Medical Center, Durham, NC, United States

**Keywords:** glomerular disease, reverse phenotype, *COL4A5* gene, IgA nephropathy, acute glomerulonephritis

## Abstract

Glomerular diseases (GDs) are a major cause of chronic kidney disease in children. The conventional approach to diagnosis of GDs includes clinical evaluation and, in most cases, kidney biopsy to make a definitive diagnosis. However, in many cases, clinical presentations of different GDs can overlap, leading to uncertainty in diagnosis and management even after renal biopsy. In this report, we identify a family with clinical diagnoses of postinfectious glomerulonephritis and IgA nephropathy in a parent and two children. Renal biopsies were initially inconclusive; however, genetic testing showed that the two individuals diagnosed at different points with IgA nephropathy carried novel segregating pathogenic variants in *COL4A5* gene. We were only able to make the final diagnoses in each of the family members after genetic testing and reverse phenotyping. This case highlights the utility of genetic testing and reverse phenotyping in resolving clinical diagnosis in families with unusual constellations of different glomerulopathies. We propose that clustering of different glomerular disease phenotypes in a family should be an indication for genetic testing followed by reverse phenotyping.

## Introduction

Glomerular diseases (GDs) account for 8–14% of all cases of chronic kidney disease (CKD) in children (1, 2). The pathogenesis of different GDs is not completely known; however, recent advances in genomics and proteomics have thrown more light on disease mechanisms. For example, it is now known that GDs that are associated predominantly with proteinuria (nephrotic syndrome) are likely due to primary or secondary injuries to the visceral epithelial cell of the glomerulus, the podocyte, hence, the name podocytopathy (3). More than 60 genes that are known to cause monogenic nephrotic syndrome are enriched in the podocyte (4). However, most cases of hereditary nephritis are due to defects in collagen alpha genes (*COL4A3, COL4A4*, and *COL4A5*) (5).

While the majority of GDs are diagnosed by standard clinical evaluation and kidney histopathology, additional investigations, such as genetic testing, may be indicated to establish diagnosis. Even when genetic etiology is established, there is considerable overlap between different GDs. For example it has been reported that up to 10% of classical hereditary podocytopathies (focal and segmental glomerulosclerosis) are due to pathogenic variants in *COL4A* genes, and pathogenic variants in these same genes have also been reported in patients with presumed IgA nephropathy (6–10). This reported phenotypic pleiotropy in genes associated with GD emphasizes the importance of genetic diagnosis and careful scrutiny of phenotype data after genetic diagnosis to ensure accurate disease classification (10). In this report, we describe a family with unusual aggregation of different GDs that was clearly defined by a combination of genetic diagnosis and careful reverse phenotyping.

## Case Description

YB is a 7-year-old male with no significant medical history who presented to the emergency department with hematuria in the context of 3-day history of fever, cough, and sore throat. He denied dysuria, epistaxis, hematochezia, or mucosal bleeding. Physical examination was notable for normal blood pressure, normal oropharyngeal appearance, and absence of peripheral edema. Urinalysis revealed 1+ protein, 3+ blood, and 1+ leukocytes. Urine protein/creatinine ratio was 1,167 mg/g. Serum creatinine was 0.5 mg/dl. Additional serum chemistries, complete blood count, C3/C4, anti-streptolysin O, and anti-DNase B were all within normal limits. Serologic testing was negative for anti-dsDNA, ANCA, and anti-GBM. Renal ultrasound revealed symmetrically large kidneys bilaterally without hydronephrosis, nephrolithiasis, or masses. He was treated for presumed UTI and referred to a pediatric nephrology clinic for follow up.

**Figure 1:**
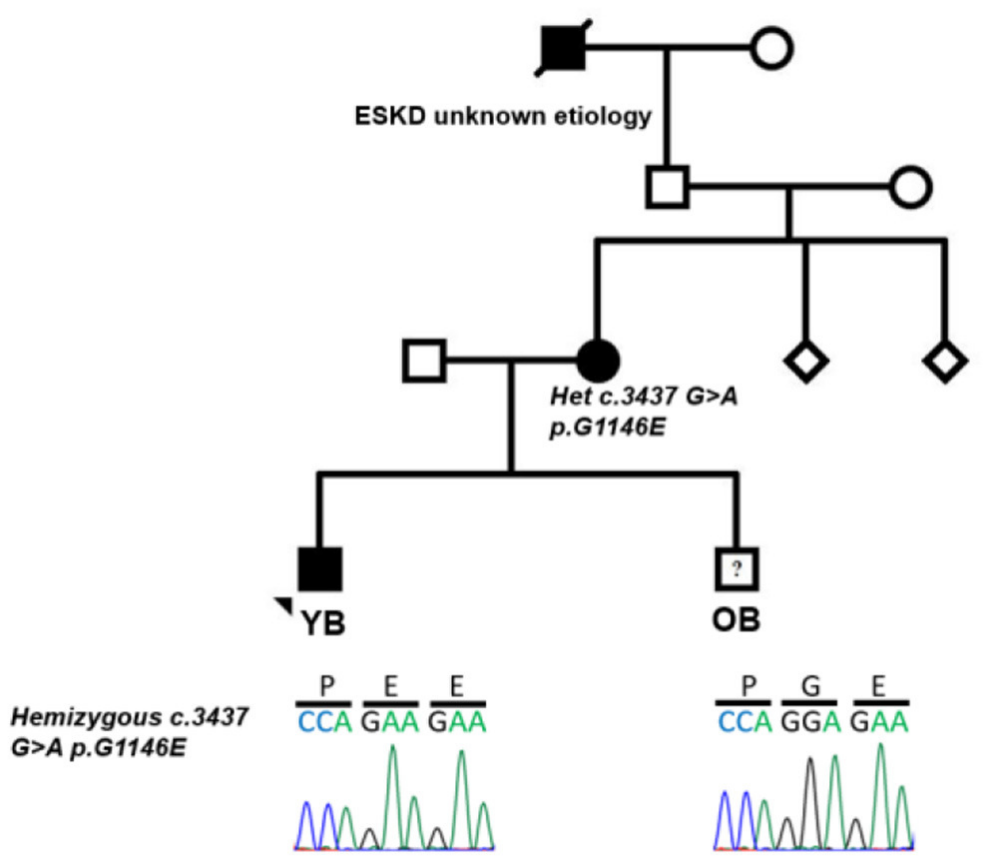
Pedigree of family with aggregation of different glomerular diseases showing segregation of c.3437 G>A p.G1146E in *COL4A5*. Filled circle and square represent affected individuals; unfilled circle, square, and diamond represent unaffected individuals.

Approximately 2 months following his initial presentation, YB had recurrence of gross hematuria concomitantly following a viral syndrome. Rapid streptococcal testing was negative. Urinalysis revealed 3+ blood and persistent subnephrotic-range proteinuria. His symptoms resolved within a week. Given the concern for postinfectious glomerulonephritis vs. IgA nephropathy, close follow-up was arranged. Over the subsequent 9 months, he continued with microscopic hematuria and low-grade proteinuria. Upon further review of family history, it was discovered that his mother had microscopic hematuria and hypertension at 9 years of age, which was thought to be due to IgA nephropathy; she was managed conservatively, and his maternal great grandfather had a history of end-stage kidney disease (ESKD) of unclear etiology (Figure 1). Given persistent low-grade proteinuria and significant family history of kidney disease, kidney biopsy and genetic testing were pursued in YB. Notably, YB had no history of hearing loss.

**Figure 2:**
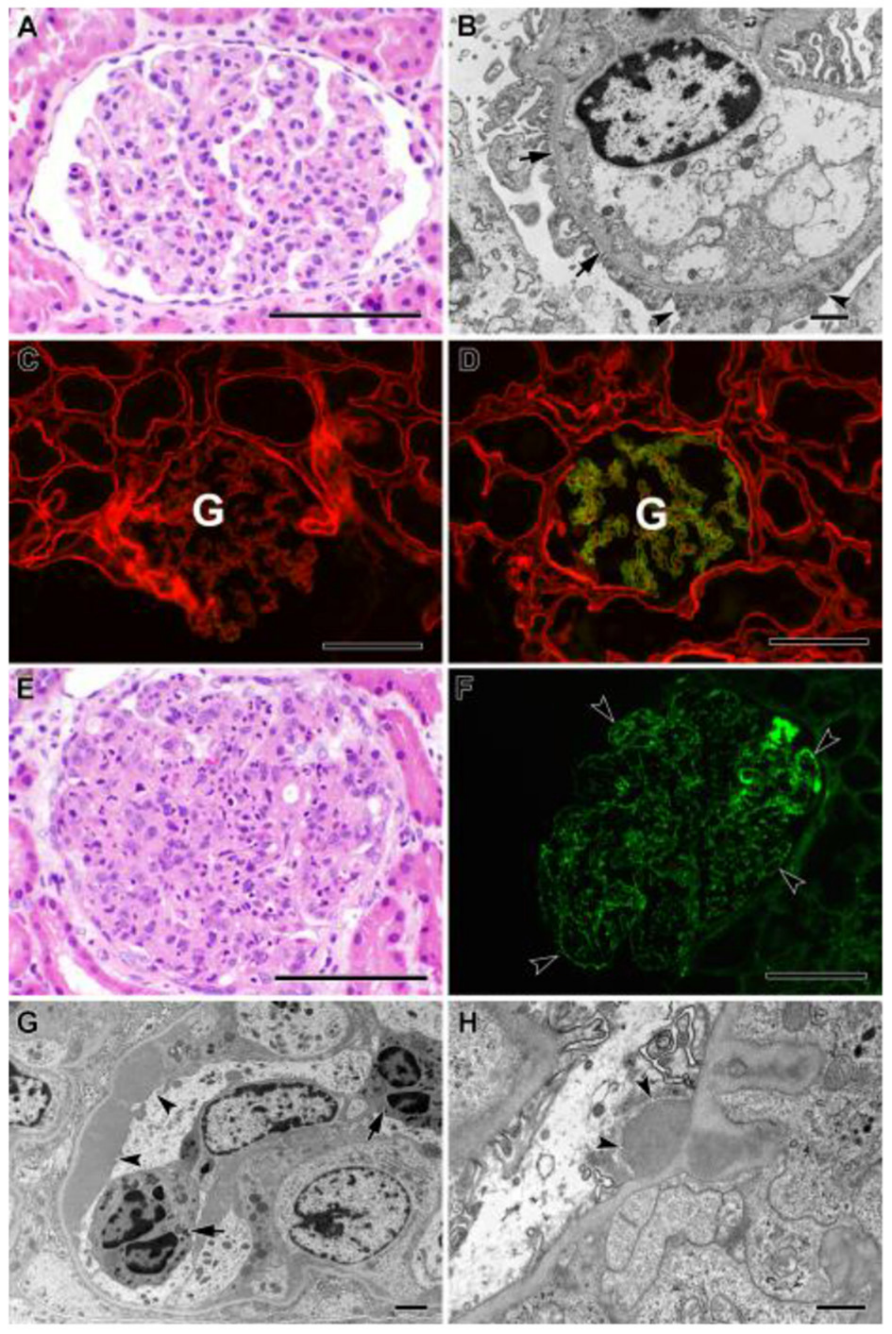
**(A–D)** Biopsy findings in patient YB. **(A)** Glomerulus from paraffin section showing minimal histologic abnormality (hematoxylin and eosin stain). Bar = 100 μm. **(B)** Electron micrograph showing mild, patchy podocyte foot process effacement (arrowheads), and mild thinning of capillary loop basement membrane without thickening or lamellation (arrows). No immune complex deposits were identified. Bar = 1 μm. **(C,D)** Two-color immunofluorescent stains of fresh frozen tissue for α2 (red) and α5 (green) chains of type IV collagen. **(C)** Patient's biopsy, showing total loss of staining for α5 chain in the glomerulus **(G)**, consistent with Alport syndrome. **(D)** Positive control tissue showing normal pattern of staining for α5 chain confined to capillaries in the glomerulus **(G)**, with more diffuse staining for α2 chain. Bars = 100 μm. **(E–H)** Biopsy findings in patient OB. **(E)** Glomerulus from paraffin section, showing global hypercellularity with abundant infiltration of neutrophils (hematoxylin and eosin stain). Bar = 100 μm. **(F)** Immunofluorescent stain of fresh frozen biopsy tissue for IgG showing extensive granular staining of capillary loops (arrowheads). Bar = 100 μm. **(G)** Electron micrograph showing large subendothelial immune complex deposit (arrowheads) and neutrophils within capillary lumen (arrows). Bar = 2 μm. **(H)** Electron micrograph showing subepithelial immune complex deposit (“hump,” arrowheads). Bar = 800 nm.

Kidney biopsy revealed rare foci of extracapillary proliferation of epithelial cells without significant mesangial or endothelial cell proliferation, necrosis, or inflammation. Mild, focal, segmental staining of mesangial areas for IgA was identified by immunofluorescence. On electron microscopy, glomerular basement membranes appeared somewhat thinned, without thickening or lamellation; there was mild podocyte foot process effacement, but no unequivocal immune complex deposits were detected (Figures 2A,B). These findings were initially reported to be most consistent with IgA nephropathy. Genetic studies were carried out on a panel of *COL4A* and FSGS genes. Results revealed two variants of uncertain significance (VUS) in *CD2AP* (exon 7 heterozygous; c.730C>T p.Pro244Ser) and *COL4A5* (exon 38 hemizygous; c.3437G>A genes (Figure 1). The *COL4A5* variant is not present in over 250,000 exomes in the Genome Aggregation Database (gnomAD) database. The variant was predicted to be damaging by three *in silico* tools. The *CD2AP* variant was found in 14 individuals in the gnomAD database (minor allele frequency: 0.00006) and was also predicted to be benign by SIFT *in silico* tools. Based on these findings, immunofluorescent staining for COL4A was performed on the previously obtained kidney biopsy and demonstrated negative staining for collagen type IV alpha 3 and 5 in the glomerular basement membranes, negative staining for alpha 5 in Bowman's capsule, and positive staining for alpha 2 in the glomerular basement membranes and mesangium, all findings consistent with *COL4A* disease (Figures 2C,D).

Based on the family history, genetic testing, and COL4A staining, YB was diagnosed with Alport syndrome due to a variant in the *COL4A5* gene. YB has no history of hearing loss and passed his hearing test in the newborn period; however, his mother was recently diagnosed with bilateral hearing loss and ongoing microscopic hematuria. We carried out genetic testing in the mother and showed that she has the same *COL4A5* variant detected in YB but not the *CD2AP* variant (Figure 1). We confirmed the *COL4A5* variant by direct sequencing.

Shortly after YB was diagnosed with Alport syndrome, his 13-year-old brother (OB) presented to the emergency department (ED) with a 2-day history of fever, poor oral intake, emesis, abdominal pain, and oliguria. History was significant for Group A streptococcal pharyngitis diagnosed 1 month prior. Physical examination in the ED was notable for hypertension, petechial rash on the face and chest, and bilateral pitting pedal edema. Renal function panel revealed hyperkalemia (7.1 mmol/l) and stage three acute kidney injury (BUN 71 mg/dl, Cr 3.1 mg/dl). Urinalysis revealed 3+ blood, 2+ protein, urine protein/creatinine ratio of 388 mg/g, low C3 complement, and normal C4 complement. Serology and infectious disease work up were all negative. A presumptive diagnosis of postinfectious glomerulonephritis was made, but a renal biopsy was performed to rule out Alport syndrome in view of the family history. Histology of the kidney biopsy done on OB showed diffuse proliferative and exudative glomerulonephritis with global finely granular and focal segmental globular IgG and C3 deposits on immunofluorescence. Electron microscopy showed extensive subendothelial immune complex deposits as well as occasional subepithelial “hump-like” deposits, consistent with postinfectious glomerulonephritis (Figures 2E–H). Alport immunofluorescence staining showed no definitive loss of the alpha 3, and 5 chains of type IV collagen. OB's acute kidney injury was managed with acute dialysis, fluid restriction, and blood pressure control. His renal function normalized. He currently has no proteinuria, and his creatinine at the last follow up was 0.6. His genetic testing did not show the *COL4A5* and *CD2AP* variant that was reported in his mother and brother (Figure 1).

## Discussion

This case highlights the utility of genetic testing and reverse phenotyping in resolving clinical diagnosis (11). While presumptive diagnoses of IgA nephropathy vs. postinfectious glomerulonephritis were considered in the proband (YB), these diagnoses were questioned in the setting of persistent proteinuria and suspicious family history; inconclusive kidney biopsy findings prompted genetic testing, which enabled the subsequent delineation of glomerulopathies in this family.

Alport syndrome (AS) is an inherited glomerulopathy characterized by hematuria, proteinuria, progressive renal failure, hearing loss, and ocular abnormalities (5). Alport syndrome is caused by mutations in type IV collagen genes (*COL4A3, COL4A4*, and *COL4A5*) (5, 12). 85% of known mutations are inherited in x-linked manner (*COL4A5*), and the remaining 15% of mutations are inherited in an autosomal recessive or autosomal dominant manner (*COL4A3* or *COL4A4* mutations) (5, 12), although it should be noted that autosomal dominant Alport syndrome is probably underdiagnosed because of its subtle phenotype in the majority of affected individuals.

A 2018 and, more recently, a 2021 consensus report suggests new classification for AS because of wide variability in its clinical phenotype and estimated that the lifetime risk for chronic kidney disease (CKD) is 100, 25, 100, and 20% for X-linked recessive, X-linked heterozygous, autosomal recessive, and autosomal dominant AS respectively (5, 13).

Our report adds to previously reported data on wide variability in the clinical manifestations of *COL4A*-associated nephropathy and significant overlap of this condition with other distinct glomerular pathologies. We and others reported that up to 10% of patients with diagnosis of familial FSGS have pathogenic variants in one of the *COL4A* genes; subsequent large studies showed that *COL4A* variants are the most common cause of CKD of unknown etiology in adults (6–8). Two studies also found pathogenic variants in *COL4A3, COL4A4, and COL4A5* in 10–20% of patients with presumed IgA nephropathy (9, 10). One approach that investigators have used to ensure accurate diagnosis of kidney disease after genetic testing is by performing reverse phenotyping after genetic testing. Reverse phenotyping is a process whereby patients and their family are reevaluated after genetic testing with the goal of segregating identified genetic variants to previously unrecognized clinical symptoms and increase the sensitivity of the genetic analysis (11). In a recent study, Landini et al. showed that reverse phenotyping was able to identify previously unrecognized clinical signs of an unexpected underlying genetic nephropathy in 28% of patients in their cohort after whole-exome sequencing (11).

The *COL4A5* variant reported in this family has not been found in patients with AS before and was initially reported as variant of unknown significance (VUS). However, further clinical evaluation and additional ancillary work-up clearly establish the pathogenicity of this variant. In addition, it also allows for accurate diagnosis and management of a sibling who presented with a completely different non-monogenic glomerular disease shortly after establishing diagnosis of AS in the proband. Our findings highlight the need for the future nephrologists to be trained in the use and interpretation of genetic testing to make accurate diagnosis of different kidney diseases. They also emphasize the need for integration of clinical, pathologic, and genomic data in the diagnosis of glomerular and other kidney diseases.

In conclusion, we propose that unusual aggregation of different glomerular diseases in a family is an indication for genetic testing followed by reverse phenotyping.

## Author Contributions

RG design the studies, participate in the recruitment of participants in the study, genetic studies, and deep phenotyping, and writing of the manuscript. RK participate in the recruitment of participants in the study, genetic studies, and deep phenotyping and writing of the manuscript. VK participate in the recruitment of participants in the study, deep phenotyping and editing of the manuscript. MS performed the genetic studies and was involved in writing of the manuscript. BL performed the genetic studies and was involved in writing of the manuscript. AB, LB, and DH generated and interpreted the renal biopsy. All three were involved in writing and editing of the manuscript. All authors contributed to the article and approved the submitted version.

## Conflict of Interest

The authors declare that the research was conducted in the absence of any commercial or financial relationships that could be construed as a potential conflict of interest.

## Publisher's Note

All claims expressed in this article are solely those of the authors and do not necessarily represent those of their affiliated organizations, or those of the publisher, the editors and the reviewers. Any product that may be evaluated in this article, or claim that may be made by its manufacturer, is not guaranteed or endorsed by the publisher.
